# Beyond pressure: intracranial compliance and retinal biomarkers in idiopathic normal pressure hydrocephalus

**DOI:** 10.3389/fneur.2026.1753259

**Published:** 2026-03-10

**Authors:** Mathias Just Nortvig, Niclas Lynge Eriksen, Jan Saip Aunan-Diop, Bjarni Johannsson, Dag Ferner Netteland, Christian Bonde Pedersen, Sune Munthe, Frantz Rom Poulsen

**Affiliations:** 1 Department of Neurosurgery, Odense University Hospital, Odense, Denmark; 2 Clinical Institute and BRIDGE (Brain Research-Inter Disciplinary Guided Excellence), University of Southern Denmark, Odense, Denmark; 3 Department of Neurosurgery, Oslo University Hospital, Oslo, Norway; 4 Faculty of Medicine, University of Oslo, Oslo, Norway

**Keywords:** A/V ratio, idiopathic normal pressure hydrocephalu (iNPH), intracranial compliance (ICC), non-invasive neuroimaging, retinal fundoscopy

## Introduction

### From pressure to compliance

Intracranial pressure (ICP) has long been regarded as the fundamental physiological variable in neurosurgical and neurocritical care. However, decades of clinical observation demonstrate that pressure alone cannot account for the clinical course of many chronic intracranial disorders.

Idiopathic normal pressure hydrocephalus (iNPH) exemplifies this paradox: patients present with gait disturbance, cognitive decline and ventriculomegaly, however, their mean ICP values often remain within the physiological range (1). The missing variable is intracranial compliance (ICC), describing the brain's ability to accommodate volume fluctuations without disproportionate increases in pressure. Reduced ICC reflects a loss of compensatory reserve, which can exist even in the presence of normal ICP. This concept reframes iNPH not as a disorder of pressure, but as a disease of compliance. Once this shift in understanding is recognized, the logical progression for clinical monitoring is to move beyond absolute ICP values and toward quantification of compliance dynamics, preferably using non-invasive techniques.

In this opinion paper, we outline how invasive monitoring data have illuminated the compliance deficit underlying iNPH, how retinal imaging may serve as an early and accessible non-invasive surrogate, and which methodological and conceptual challenges must be addressed before such biomechanical metrics can be translated into routine clinical use.

## Subsections

### iNPH as a disease of compliance

**Figure 1:**
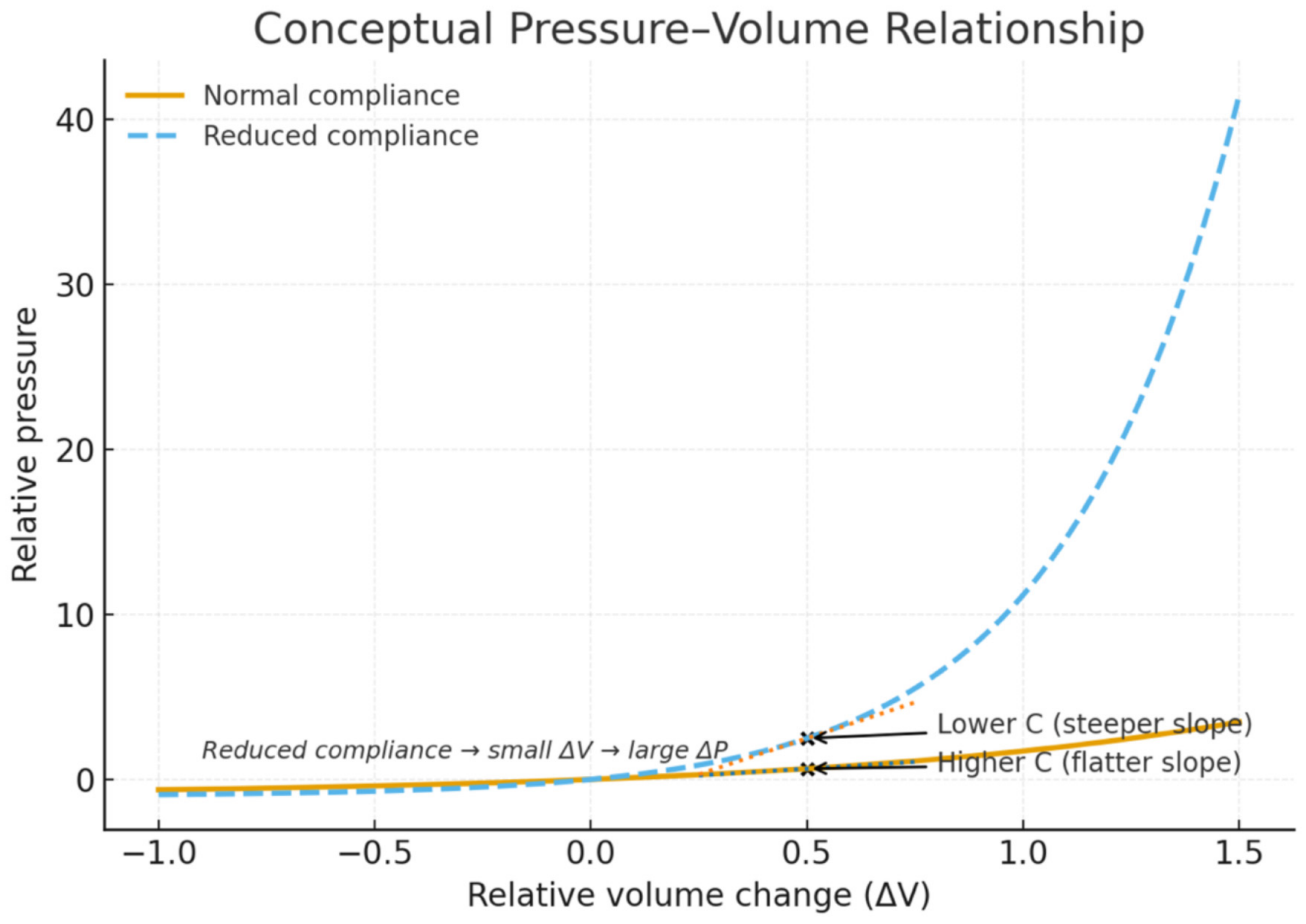
Conceptual pressure–volume relationship. Schematic illustration of intracranial pressure–volume dynamics under conditions of normal (yellow) and reduced (blue) intracranial compliance. A steeper slope indicates lower compliance, where small volume increases (ΔV) produce large pressure changes (ΔP).

The Monro–Kellie doctrine asserts that the total intracranial volume, consisting of brain tissue, blood, and cerebrospinal fluid, remains nearly constant (CSF) (2, 3). In healthy individuals, these compartments dynamically compensate for one another. As arterial inflow increases, CSF redistributes into the spinal canal and venous outflow rate accelerates. This non-linear capacity defines ICC, mathematically, ΔV/ΔP (Figure 1).

In iNPH, ICC gradually deteriorates because of pathophysiological processes, while shunt treatment may partially restore some of this compensatory capacity (4, 5). Invasive infusion studies and waveform analysis have demonstrated that patients with iNPH exhibit a steeper pressure-volume relationship and reduced buffering capacity despite normal or near normal mean ICP values (6, 7). Several mechanisms have been implicated in this compliance failure. Impaired cerebrospinal fluid absorption at the arachnoid granulations increases the effective outflow resistance of the intracranial system, while abnormalities into the venous drainage, including dural sinus stenosis, elevated venous pressure, and altered jugular outflow, further limit the capacity to accommodate pulsatile volume changes (8, 9). Together these processes shift the operation point of the intracranial pressure-volume curve toward a steeper region, where small increases in intracranial volume produce disproportionately large pressure pulsations rather than sustained elevations in mean ICP (10). This pathophysiological explanation provides a mechanistic explanation for how iNPH can manifest with preserved mean ICP but markedly reduces ICC, and why shunt treatment may improve symptoms by restoring pressure-volume buffering rather than simply lowering static pressure. Retinal venous caliber has been shown to respond to changes in intracranial CSF dynamics in clinical settings, including CSF diversion studies, supporting the sensitivity of the retinal venous compartment to upstream pressure and compliance changes. Given the shared venous drainage between the retinal and the intracranial venous system, impaired ICC with elevated venous outflow resistance provides a plausible mechanism for retinal venous distention and a reduced A/V ratio. Nevertheless, the A/V ratio should be regarded as a physiologically motivated surrogate rather than a validated biomarker of ICC and its clinical utility requires further prospective validation.

**Figure 2:**
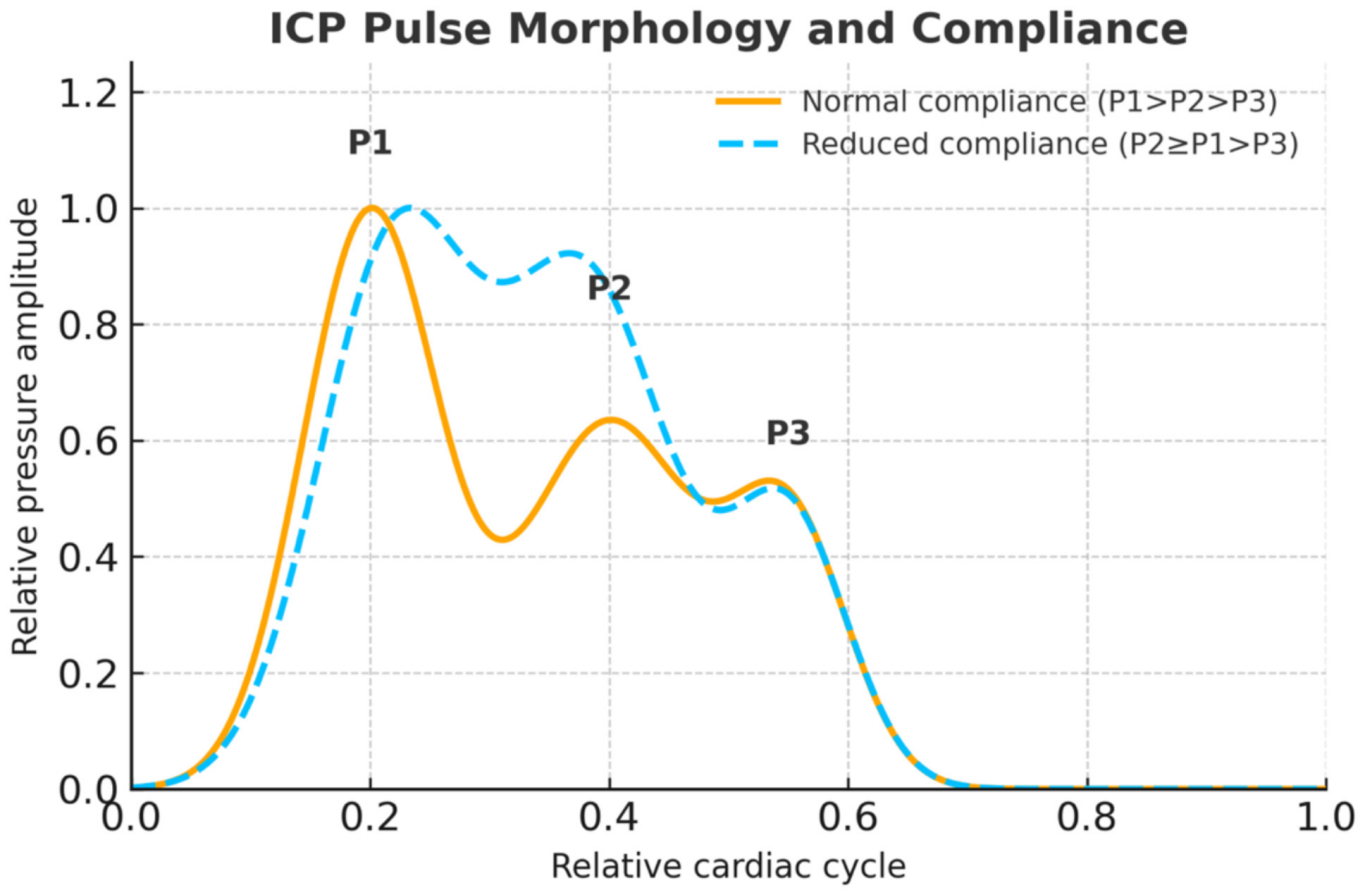
ICP pulse morphology and intracranial compliance. Schematic representation of the intracranial pressure (ICP) waveform across the cardiac cycle, illustrating the characteristic peaks: P1 (percussion wave), P2 (tidal wave), and P3 (dicrotic wave). Under normal compliance (solid orange line), P1 amplitude exceeds that of P2, reflecting preserved intracranial elasticity. When compliance is reduced (stippled blue line), the amplitude of P2 approaches that of P1, and may eventually exceed it, indicating impaired compensatory capacity.

On waveform analysis, reduced compliance manifests as an elevation of the tidal (P2) component relative to the percussion (P1) wave, indicating a diminished ability of the intracranial compartment to accommodate arterial pulsations (2, 11) (Figure 2). This P2–P1 relationship has been observed with invasive monitoring both during infusion tests in iNPH and in acute pathologies such as traumatic brain injury. Absolute threshold ratios do however vary substantially between conditions, devices and analytical methods (2). Therefore, while a higher P2 relative to P1 is a consistent physiological signature of impaired compliance, fixed cut-offs should be interpreted cautiously and always within the specific diagnostic and technical context (2, 12).

### Lessons from invasive monitoring

Invasive ICP monitoring has provided the most detailed insights into ICC, but its clinical application is limited by practical availability, especially in more chronic conditions. In iNPH, invasive studies have shown that pressure–volume indices and waveform morphology change dynamically during CSF infusion or drainage, correlating with shunt responsiveness (13, 14). The methods yield precise, real time physiological information but suffer from several shortcomings. First, the invasive nature of these procedures restricts their use to specialized units and excludes certain patient populations. Second, while invasive ICP monitoring devices including intraparenchymal probes and external ventricular drains allow for continuous monitoring, their use is inherently time limited and carry risks of infection, intracranial hemorrhage and device malfunction. As a result, they are unsuitable for long term or ambulatory assessment of intracranial dynamics. Third, different analytical methods for quantifying ICC remain insufficiently standardized across centers, which limits reproducibility and comparison between studies. This variability is an expression of the current limitations in the quality of evidence, concerning iNPH diagnostics. Despite these issues, invasive recordings remain the gold standard for validating any non-invasive surrogate. The initial challenge, therefore, is not to outright replace invasive methods, but to identify non-invasive signatures that can capture ICC behavior.

### The eye as a window to compliance: physiological rationale

The retina and brain share embryological origin, vascular regulation and venous drainage (15, 16). The central retinal vein drains via the superior ophthalmic vein into the cavernous sinus, which communicates directly with the intracranial venous system (17). This anatomical continuity means that alterations in intracranial venous pressure and compliance can be transmitted to the retinal circulation. With reduced ICC, the resistance to venous outflow from the retina rises, leading to distension of retinal venules, whereas retinal arterioles remain comparatively stable due to the process of autoregulation (18). With declining ICC, the intracranial venous system operates at a higher filling pressure, effectively increasing venous preload. This leads to a higher pressure gradient for venous return, thereby increasing outflow resistance from the retinal venules. The resulting venous congestion leads to distension of retinal venules, whereas arteriolar caliber remains comparatively stable owing to autoregulatory control. This process can be expressed as the retinal arterial/venous (A/V) ratio, reflecting the hemodynamic consequences of impaired venous drainage and reduced ICC. Unlike other static parameters aiming to assess ICP non-invasively, such as optic nerve sheath diameter (ONSD), the A/V ratio captures the microvascular consequence of impaired buffering capacity, integrating both hemodynamic and structural effects. However, the relationship is likely modulated by systemic factors such as blood pressure, intraocular pressure and carbon dioxide levels, as well as regional variations in compliance across brain compartments (19). Consequently, the A/V ratio might be viewed as an indirect yet physiologically grounded indicator of ICC rather than a surrogate for absolute pressure.

## Discussion

### Retinal A/V ratio as a compliance marker

A pilot study of patients undergoing evaluation for iNPH suggested that the A/V ratio was significantly lower in patients who were subsequently diagnosed with iNPH compared to those classified as non-iNPH (mean 0.78 vs. 0.86, *p* = 0.02) (20). We have also shown that the A/V ratio is inversely correlated to the ICP, when ICP is above 15–20 mmHg (21–24). Despite comparable baseline ICP values, the difference in retinal vessel morphology indicates that the A/V ratio may reflect compliance related venous changes rather than absolute ICP. Diagnostic performance analysis yielded an AUC of 0.75, with a sensitivity of 88%, and specificity of 50% at an A/V cutoff of 0.86. This profile suggests high screening potential but limited diagnostic precision. Importantly, there was a tendency toward lower preoperative A/V ratios among shunt responsive patients, although this trend did not reach statistical significance (20). The observation is consistent with the hypothesis that A/V ratio could serve as a potential dynamic marker of compliance restoration following CSF shunting. While encouraging, these findings require cautious interpretation. The study's high image exclusion rate (26 of 50 patients) introduces potential selection bias and the absence of confounder adjustment leaves a possibility of influence from systemic vascular disease, hypertension and diabetes which are all known to alter retinal microvasculature (25). Nevertheless, the consistent directionality across groups underscores the physiological plausibility of retinal venous morphology as a marker of impaired ICC.

### Positioning among non-invasive ICP techniques

Current non-invasive ICP modalities, including ONSD ultrasound, transcranial Doppler (TCD)–derived pulsatility indices and tympanic membrane displacement, provide valuable but incomplete surrogates of intracranial dynamics (26–29). Each targets a specific component of the intracranial system. ONSD reflects transmission of ICP in the CSF along the optic nerve, TCD indices mirror cerebrovascular resistance and flow pulsatility, and tympanic membrane displacement captures CSF pressure transmission through the cochlear aqueduct. While these methods can infer pressure trends, they primarily describe static or flow related phenomena rather than the underlying pressure–volume relationship that defines ICC. The closest analog to A/V ratio–based assessment is the Brain4Care device, which uses cranial deformation sensors to record ICP waveforms and extract the P2/P1 ratio as a marker of reduced compliance and risk of intracranial hypertension (30).

Previous OCT-studies have reported choroidal structural and microvascular alterations in iNPH (31, 32), findings that are compatible with a venous outflow and compliance-based disease model. These observations support the concept that ocular vascular compartments beyond the retina may be influenced by changes in intracranial dynamics. However, the present Opinion focuses on fundus-based retinal imaging due to its feasibility within neurological clinical workflow and its potential for bedside application outside ophthalmological settings. Retinal imaging, by contrast, introduces a complementary paradigm. Rather than inferring compliance from skull or flow dynamics, it directly visualizes the microvascular end organ response to altered intracranial hemodynamics and compliance. The venous compartment of the retina in particular, appears sensitive to early compliance loss, showing morphological changes even before overt pressure elevation. This may provide a window into the vascular consequences of impaired ICC, potentially bridging physiological and morphological assessment.

Nevertheless, like other non-invasive methods, retinal imaging is face by some limitations, including image quality variability, dependence on pupil size and illumination and the absence of standardized diagnostic thresholds. Overcoming this will require both technical refinement and harmonized calibration across devices and centers.

Integration represents the most promising path forward. Combining retinal imaging with ONSD ultrasound, TCD metrics and waveform-based devices into a multimodal compliance index could yield a more holistic and reliable measure of ICC. Incorporating artificial intelligence, neural network models could analyze retinal images beyond the A/V ratio to identify additional objective markers of elevated ICP, thereby refining and optimizing the method. Such an approach would emphasize synergy rather than competition between modalities, leveraging the physiological specificity of each and moving the field closer to its long-standing objective, the “holy grail” of accurate, non-invasive ICP, and ICC monitoring.

### Methodological barriers and how to overcome them

The methodological limitations identified in our study are informative for both our own future work and related research in the field. First, image quality and selection bias remain major obstacles. Many recordings in our studies on A/V ratio were excluded due technical factors including small pupils and suboptimal focus, emphasizing the need for standardized imaging conditions, improved optical hardware and clear protocols for quality assurance. Second, confounding by systemic vascular factors must be addressed through comprehensive multivariable analysis and inclusion of vascular comorbidity indices. Without such adjustment, specificity for cerebral pathology will remain uncertain. Third, the currently used AI algorithm for detecting impaired ICC has not yet achieved external validity across diverse patient populations. Future AI models must include automated quality gating, calibration against invasive data, and interpretability metrics that prevent algorithmic bias. Looking ahead, AI may also facilitate multimodal integration, linking retinal imaging with complementary non-invasive modalities such as ONSD assessment, TCD, or systemic physiological parameters.

Finally, compliance is inherently a dynamic property. Future studies should employ timed pairing between fundus imaging and invasive pressure waveforms, allowing mapping of retinal changes along the pressure–volume curve. Such synchronization could transform static imaging into dynamic compliance assessment, bridging the gap between physiological understanding, and technological application.

From an implementation perspective, progress toward clinical translation will require a stepwise validation and standardization strategy. Image acquisition protocols should define minimal quality criteria for optic disc visibility, vessel sharpness, and illumination stability, with automated quality control to ensure reproducibility across devices and operators. Confounders known to influence retinal vasculature, including age, systemic vascular disease, diabetes, ocular pathology, and vasoactive medications, should be addressed through predefined exclusion criteria or incorporated as covariates in multivariable models.

Confounders that may influence retinal vessel caliber should be handled using a prespecified strategy that distinguishes true confounding from potential over adjustment. Core vascular covariates can be addressed through study design and sensitivity analyses, while variables closely linked to intracranial physiology or disease severity should be treated cautiously to avoid adjusting away the signal of interest.

Analytical pipelines should be standardized with transparent reporting of vessel segmentation methods, measurement locations, and A/V ratio computation to enable comparison across studies. Finally, validation should follow a staged pathway from pilot feasibility studies to cross-sectional association with invasive or waveform-based compliance measures, to longitudinal and multicenter replication using clinically meaningful reference outcomes.

### A roadmap for compliance oriented monitoring

Future progress depends on large, prospective studies explicitly designed to evaluate ICC, rather than pressure alone. Key priorities include prospective, multicenter recruitment with standardized imaging and invasive reference data, as well as pre- and post-shunt assessments in iNPH to capture dynamic changes in both retinal and systemic compliance markers. Emphasis could be placed on integrating magnetic resonance elastography (MRE), which quantifies regional brain tissue stiffness and thereby complements optical and hemodynamic measures of compliance (4, 33). Combining retinal multimodal imaging with MRE could enable a more comprehensive characterization of ICC variation across compartments and over time.

The development of composite compliance indices that merge complementary signals could advance monitoring from a single parameter approach toward a multidimensional assessment of intracranial dynamics. Through these coordinated efforts, non-invasive monitoring may evolve from a proof-of-concept toward genuine clinical utility.

## Conclusion–toward a compliance based paradigm

The evidence from invasive and retinal studies points toward a unifying insight that, in iNPH, it is ICC rather than pressure that defines the disease (34–37). Reduced compliance appears to underlie the triad of symptoms without an increase in ICP. The retinal A/V ratio may offer a physiologically plausible and fully non-invasive view into this process, showing measurable alterations in patients with impaired compliance and a tendency toward normalization among those who respond to shunt surgery (20).

Future non-invasive technologies should therefore be assessed not only for their ability to estimate pressure but for how accurately they capture ICC dynamics. A sustained focus on ICC rather than pressure has the potential to reshape the field of neuromonitoring, enabling earlier detection of decompensation, more individualized treatment planning and ultimately better outcomes across both acute and chronic neurological disorders. This approach may also be transferable to patients with traumatic brain injury, where monitoring compliance could allow identification of increasing ICP before it clinically manifests.
